# Guideline on multimodal rehabilitation for patients with post-intensive care syndrome

**DOI:** 10.1186/s13054-023-04569-5

**Published:** 2023-07-31

**Authors:** Caroline Renner, Marie-Madlen Jeitziner, Monika Albert, Sabine Brinkmann, Karin Diserens, Imanuel Dzialowski, Maria-Dorothea Heidler, Martina Lück, Ricki Nusser-Müller-Busch, Peter S. Sandor, Andreas Schäfer, Bettina Scheffler, Claus Wallesch, Gudrun Zimmermann, Peter Nydahl

**Affiliations:** 1 Department of Neurology and Neuro-Rehabilitation, Herz-Kreislauf-Zentrum, Klinikum Hersfeld-Rotenburg GmbH, Rotenburg a. F., University of Leipzig, Leipzig, Germany; 2 Department of Intensive Care Medicine, Inselspital, Bern University Hospital, University of Bern, Bern, Switzerland; 3 Institute of Nursing Science, Department of Public Health, Faculty of Medicine, University of Basel, Basel, Switzerland; 4 Department of Neurology, Rehabilitation ZURZACH Care, Baden, Switzerland; 5 University of Applied Sciences, Osnabrück, Germany; 6 Department of Clinical Neurosciences, University Hospital, Lausanne, Switzerland; 7 ELBLAND Neuro-Rehabilitation Center Grossenhain, Academic Teaching Hospital Technical University Dresden, Dresden, Germany; 8 Brandenburg Klinik, Bernau, Germany; 9 Fachklinik Bad Heilbrunn, Heilbrunn, Germany; 10 Berlin, Germany; 11 Department Neurology and Psych. ZURZACH Care, Bad Zurzach, Switzerland; 12 Asklepios Center for Further Education in Intensive Care - and Anaesthesia Nursing North Hesse, Frankfurt, Germany; 13 Brandenburg University of Technology Cottbus-Senftenberg, Senftenberg, Germany; 14 BDH-Hospital Elzach - Center for Neurorehabilitation and Intensive Care, Elzach, Germany; 15 IB University of Health and Applied Social Science Berlin, Hamburg, Cologne, Stuttgart, Munich, Germany; 16 Nursing Research, University Hospital of Schleswig-Holstein, Arnold-Heller-Str. 3, 24105 Kiel, Germany; 17 Institute of Nursing Science and Development, Paracelsus Medical University, Salzburg, Austria

**Keywords:** Critical care, Guidelines, Intensive care, Physical therapy, PICS, Post-intensive care syndrome, Psychological therapy, Rehabilitation

## Abstract

**Background:**

Intensive Care Unit (ICU) survivors often experience several impairments in their physical, cognitive, and psychological health status, which are labeled as post-intensive care syndrome (PICS). The aim of this work is to develop a multidisciplinary and -professional guideline for the rehabilitative therapy of PICS.

**Methods:**

A multidisciplinary/-professional task force of 15 healthcare professionals applied a structured, evidence-based approach to address 10 scientific questions. For each PICO-question (Population, Intervention, Comparison, and Outcome), best available evidence was identified. Recommendations were rated as “strong recommendation”, “recommendation” or “therapy option”, based on Grading of Recommendations, Assessment, Development and Evaluation principles. In addition, evidence gaps were identified.

**Results:**

The evidence resulted in 12 recommendations, 4 therapy options, and one statement for the prevention or treatment of PICS. Recommendations: early mobilization, motor training, and nutrition/dysphagia management should be performed. Delirium prophylaxis focuses on behavioral interventions. ICU diaries can prevent/treat psychological health issues like anxiety and post-traumatic stress disorders. Early rehabilitation approaches as well as long-term access to specialized rehabilitation centers are recommended. Therapy options include additional physical rehabilitation interventions. Statement: A prerequisite for the treatment of PICS are the regular and repeated assessments of the physical, cognitive and psychological health in patients at risk for or having PICS.

**Conclusions:**

PICS is a variable and complex syndrome that requires an individual multidisciplinary, and multiprofessional approach. Rehabilitation of PICS should include an assessment and therapy of motor-, cognitive-, and psychological health impairments.

**Supplementary Information:**

The online version contains supplementary material available at 10.1186/s13054-023-04569-5.

## Key points

- It is important to assess physical, cognitive, and psychological health functions of critically ill patients at risk for developing post-intensive care syndrome (PICS) during their stay in the ICU, their acute and rehabilitative inpatient and outpatient care.
- Physical health and motor function can improve through early mobilization, physical therapy, additional ergometer training (cycling) and neuromuscular stimulation.
- Before initiating oral nutrition, a standardized swallowing test should rule out risks of dysphagia and aspiration.
- Psychological health can improve through integrating relatives into care, keeping ICU diaries, and psychological care.
- Cognitive health can improve with prevention of delirium, early multimodal treatment of delirium, and/or attention training.
- Health care professionals must be sensitized and trained to recognize PICS.

**Supplementary Information:**

The online version contains supplementary material available at 10.1186/s13054-023-04569-5.

## Background

**Fig. 1:**
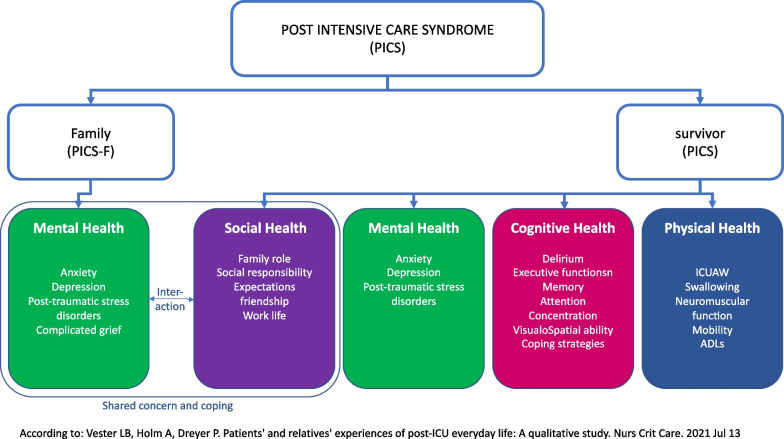
Impact of the post intensive care syndrome

Modern intensive care medicine enables more critically ill patients to survive life-threatening critical conditions. Critical illness can result from surgery, trauma, infection, or an exacerbation of a medical condition and results in malfunction of at least one organ, requiring medical, nursing, therapeutic, psychological, social, and/or technical support [1]. This support is usually provided in an Intensive Care Unit (ICU) but does not always restore health. Although survival rates are considered benchmarks of intensive care, ICU survivors are faced with increased morbidity, rehospitalization, and mortality as well as a lasting decline in health-related quality of life and participation in society [2]. The symptoms and outcomes experienced by critically ill patients are subsumed under the term post-intensive care syndrome (PICS) [3, 4]. PICS consists of a neurologically heterogenous complex of impairments which can be observed in critically ill patients after treatment in an ICU. The syndrome is characterized by new or increased impairments of physical cognitive, and/or psychological functions that outlast the stay in hospital. PICS is present if one or more of the following domains of function is impaired [3]. Cognitive impairments present as delirium and deficits of attention, memory, executive functions, and visuospatial perception. Psychological impairments consist of depression, anxiety disorders, and post-traumatic stress disorder (PTSD). Physical impairments, often summarized as “intensive care unit-acquired weakness” (ICU-AW), include neuromuscular functions of swallowing, breathing, mobility, and personal autonomy [5]. Severe axonal critical care neuropathies can result in prolonged periods of convalescence and incomplete recovery. Impairment of one function may result in impairment of another; for example, depressive symptoms may lead to a reduction of physical health [6, 7] or cognitive function [7, 8]. Symptoms of PICS may appear as early as 24 h after admission to an ICU and may persist for 5–15 years after discharge [2]. Symptoms of all three domains may emerge during any phase of the critical illness, acute, early or late. Symptoms of PICS are not specific to certain phases of critical illness. The risk to develop PICS is multifactorial; risk factors can contribute before (e.g., frailty, preexisting functional impairments), during (e.g., sedation, duration of delirium, sepsis, acute respiratory distress syndrome), and after (e.g., early symptoms of anxiety, depression, or post-traumatic stress disorder) staying in the ICU. The family can be affected, too, leading to a complex, interacting phenomenon (Fig. 1) [9].

The reported prevalence of PICS varies, due to different study populations, diagnostic criteria, or times of assessment. It has been reported that 64% and 56% of ICU survivors are impaired in a least one of the three levels of function at 3 and 12 months after discharge, respectively [10]. For impairment of at least two levels of function, reported prevalences are 25% after 3 months and 21% after 12 months. Impairments in all three levels affect 6% after 3 months and 4% after 12 months [10]. Impairments of neuromuscular functions after acute respiratory distress syndrome (ARDS) are reported in at least 25% [11] and 38–40% at the time of discharge [5, 12, 13]. The prevalence of cognitive impairments in ICU-survivors is reported as 25–40% after 3 months [14]. Reported prevalences for anxiety, depression, and PTSD at 12 months after discharge are 38%, 32%, and 18% respectively [15–18]. Given the high prevalence of PICS worldwide, guidelines for rehabilitation of critically ill patients with PICS are urgently needed. Therefore, the objective of this work was to develop a multidisciplinary and multiprofessional guideline for assessing, preventing, and treating patients affected by PICS, to improve their physical, cognitive, and psychological health. This guideline intends to promote clinical decisions and standards of care in order to improve the outcomes of adult patients at risk for developing or affected by symptoms of PICS.

## Methods

According to the system of the Association of the Scientific Medical Societies in Germany (AWMF) [19], guidelines are classified in four ranks from S1 to S3, with S3 having the highest quality level. The purpose of the S2e and S3 guidelines is to convey recommendations for clinical practice based on a comprehensive, systematic search for and a critical assessment of the available evidence. The present guideline is classified “S2e” and was coordinated by the German Society for Neurorehabilitation (DGNR). The methodological approach for the development of this guideline followed the requirements of evidence-based medicine, defined as the standard by the AWMF.

A multidisciplinary Task Force for Rehabilitation of the post-intensive care-syndrome was formed in December 2019 aiming to develop a guideline for the therapy of PICS based on the best current evidence.

**Fig. 2:**
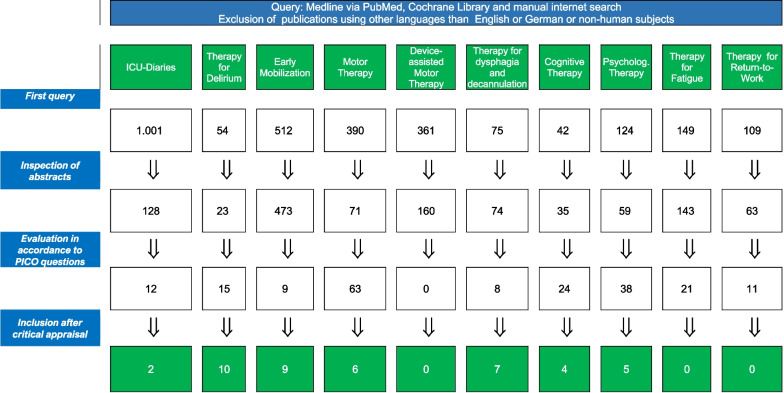
Summary of the literature search according to the 10 research questions (search period: January 2009 to December 31, 2021)

The group applied a structured, evidence-based approach to address 10 research questions that served as the basis for each recommendation and supporting rationale. The 10 research questions of pertinent importance for the therapy of PICS-related symptoms were identified by the multidisciplinary Task Force. For each research question a subgroup was formed, which developed their own search strategy according to the PICO-Scheme: Patient population (adult patients that exhibited at least one symptom in one of the three domains after critical illness and/or 48 h stay on the ICU, any gender), therapeutic intervention, comparison of intervention with no intervention or standard therapy and outcome. The 10 research questions are introducing each recommendation and are summarized in Fig. 2. The current author group includes representatives of the Swiss Society of Neurorehabilitation (SGNR) and seven relevant multidisciplinary German professional societies (DBL, DGF, DGP, DGPTW, DVE, GNP) and an organization of persons concerned (BDH) (Additional file 1: Table S1). The intended guideline audience includes all health care professionals involved in the care, diagnosis, and therapy of critical ill patients before, during, and after a treatment on ICU including physicians, nurses, therapists, and others. Furthermore, patients, families, and clinical or institutional leaders/administrators are targeted readers.

Boolean operators, medical subject headings (MeSH) and key terms were applied to structure each literature search. Searches were limited to a human patient population defined by the search terms, publications in English or German, and the time of publication from January 2009 to December 2021. Case reports or RCT with less than 10 participants were excluded). Comprehensive, structured, computer-based literature searches were performed using the indexed online database MEDLINE/PubMed, supplemented by screening of reference lists of relevant publications. The aim of each search strategy was to identify randomized controlled trials (RCTs), systematic reviews, meta-analyses or other guidelines that addressed the respective research question of each subgroup. In case of absence of high-quality scientific support, no recommendations were derived. Abstracts identified by each search strategy were screened by at least two authors and, if considered relevant, full publications were evaluated by the entire subgroup. Evaluation of literature chosen for citation in the guideline was performed according to the 2011 Oxford Centre for Evidence-Based Medicine (OCEBM) working group levels of evidence (Table 1) [20].

**Table 1:** Gradation of evidence and recommendations as an expression of the degree of certainty/uncertainty of the knowledge base for the respective recommendations

Level of evidence	According to OCEBM 2011	Symbol
Evidence level 1	Systematic review of randomized controlled studies	1
Evidence level 2	Randomized controlled study or observational study with dramatic effect	2
Evidence level 3	non-randomized controlled cohort study	3
Evidence level 4	case series, case-control studies, or historically controlled studies	4
Evidence level 5	Pathophysiological-mechanistic arguments	5

**Quality of evidence**	**In accordance to GRADE**	
High quality	Further research is unlikely to affect our confidence in the estimation of the (therapeutic) effect	
Medium quality	Further research is likely to affect our confidence in the estimation of the (therapeutic) effect and may alter the estimate	
Low quality	Further research will most likely influence our confidence in the estimation of the (therapeutic) effect and will probably change the estimate	
Very low quality	Any estimation of the (therapy) effect is very uncertain	

Recommendation grade	Wording	Symbol
Strong recommendation	Ought to/ought not to	A/A–
Recommendation	Should/should not	B/B−
Therapy option	Can be considered	0

Further, each source (original papers, systematic reviews, and meta-analyses) was evaluated regarding (1) classification of evidence level (OCEBM), its methodology (the validity), (2) the conclusions of the results were summarized, and (3) recommendations from the individual sources were derived. In a next step (4), data from all sources on a specific Research Question underwent a summarized assessment (quality of evidence in accordance to Grading of Recommendations, Assessment, Development and Evaluation (GRADE) Working Group (Table 1) [19, 21]. This reflected the confidence in the estimation of the effect strength of a therapy. The final assessment of quality was essential for deriving a recommendation. Finally, (5) clinical relevance, health benefits, patients’ preferences as known from stakeholders and the literature, side effects, and risks were considered when formulating and grading the recommendation. The letter associated with each recommendation reflects the strength of the recommendation by the author group (Table 1).

The guideline development involved 22 remote (internet-based) meetings and in-between extensive electronic communication. In December 2019, the authors participated in a web conference during which the research questions to be addressed in the guideline were defined and subsequently subgroups for each research question were assembled according to proficiency of the respective authors. Screening and evaluation of abstracts and full publications identified by the structured searches and formulation of draft recommendations and rationales was performed by the corresponding subgroups (Fig. 2). Each chapter pertaining to the respective research question was reviewed by the assigned subgroup and afterwards by the entire task force. The wording of each recommendation was finalized through the entire task force. Following revisions and approval by the task force, the manuscript was approved by the several endorsing Swiss and German societies between August and October 2022 after minor revisions. An update of this guideline is planned at the latest in four years for October 2027. The methodology applied was in keeping with the AGREE Reporting Checklist in order to control for the quality of the present guideline, the complete AGREE Reporting Checklist is reported in the digital supplement (Additional file 1: Table S2: AGREE Reporting Checklist [22]).

## Results

In total, one statement and 12 recommendations, and four therapy options for the rehabilitation of critically ill patients with PICS could be identified from the literature (Table 2). The recommendations are categorized by the three domains of impairment: Physical Rehabilitation, Cognitive Rehabilitation, and Psychological Rehabilitation.

**Table 2:** Summary of the statement, 12 recommendations, and four therapy options for the rehabilitation of critically ill patients with post-intensive care syndrome

Statement	
It is important to screen critically ill patients with a length of stay ≥ 48 h for risk factors to develop PICS and symptoms of PICS during the stay in intensive care, after discharge, during and at the end of rehabilitation, as well as in out-patient care. The choice of the optimal assessment depends on various factors such as the phase of the disease, the setting, the symptomatology, risk factors of the patient and the availability of further diagnostics	
*Recommendations and therapy options for PICS Rehabilitation*	
Rehabilitation of physical health	
1. Early mobilization ought to be started within the first few days in the ICU, adapted to the patient's resilience and general condition. (A)	
2. Supplemental use of ergometers (bed cycling) in addition to early mobilization can be considered. (0)	
3. Wheelchair cycle ergometer training can be used in addition to the standard physical therapy to improve muscle strength and cardiovascular fitness. (0)	
4. Strength training can be used as an adjunct to standard physical therapy to increase walking speed. (0)	
5. Electrical stimulation of the ventral thigh musculature can be used to strengthen the muscles. (0)	
6. Training of the inspiratory muscles using an inhalation trainer should be used to increase the strength of the inspiratory muscles and the quality of life in the short term as an adjunct to standard physical therapy. (B)	
7. As dysphagia is frequent in patients with tracheostomy, standardized swallowing assessment should be performed before oral nourishment is initiated. (B)	
Rehabilitation of cognitive health	
8. Computer-based learning of attention functions and/or therapy aiming at improvement of cognition should be performed with critically ill patients and in further rehabilitation. (B)	
9. Interventions for delirium prophylaxis ought to include multimodal sensory, cognitive and emotional stimulation (mobilization, purposeful stimulation and engagement, aids for orientation, contact to family members). (A)	
10. Interventions for stress reduction (pain, anxiety, sleep, noise), improvement of communication and family care should be applied. (B)	
11. A prophylactic treatment with Haloperidol for ventilated patients should not be implemented, as there is no effect in comparison to placebo regarding the incidence, severity, duration or outcome of delirium. (B-)	
Rehabilitation of psychological health	
12. Critically ill patients with adaptation disorders such as anxiety and depression benefit from psychological interventions. These should be offered already in the ICU and/or early rehabilitation and if possible also to relatives. (B)	
13. Post-traumatic stress reactions should be treated by interventions such as psychoeducation and psychotherapy. (B)	
14. Access to professional support and aftercare should be offered in the first 12 months after discharge aiming at mental stabilization. (B)	
15. ICU diaries ought to be implemented for reducing the risks of symptoms of anxiety, depression, and PTSD in critically ill patients after discharge from the ICU. (A)	
16. In post-ICU care, ICU diaries ought to be worked on with health care professionals. (A)	

Grade of recommendations: (0): therapy option, can be considered; (B)/(B−): recommendation, should, should not; (A)/(A−): ought to ought not to

### Statement: Diagnosis of PICS

Since no systematic literature review has been performed regarding the diagnosis of PICS, one statement instead of one recommendation has been developed according to good clinical practice.

*Research question* Which assessments can be used to diagnose and predict PICS, to recommend therapeutic interventions, and to report related progress?

*Statement 1* It is important to screen critically ill patients with a length of stay ≥ 48 h for risk factors for developing PICS and symptoms of PICS during the stay on ICU, after discharge, during and at the end of rehabilitation, and in out-patient care. The choice of the optimal assessment depends on various factors such as the phase of the disease, the setting, the symptomatology and risk factors of the patient, and the availability of further diagnostics.

*Rationale* To determine the presence and effects of PICS, many different assessments are available at the body function level to assess physical, cognitive, and psychological functions, as well as at the activity and participation level. Different approaches to assess the long-term outcome of critically ill patients include diagnostic follow-up studies [23, 24], Delphi consensus statements including former critically ill patients and relatives [12, 25], patient questionnaires [26], combined assessments pre-, peri-, and post ICU [27]. To prevent, reduce, or treat the typical symptoms of PICS, those at risk for the development of PICS should be identified as early as possible in the ICU or in early rehabilitation by means of sensorimotor, cognitive, and psychological assessments. Sensorimotor, cognitive, and psychological assessments should be repeated after discharge from the hospital or transfer to rehabilitation, and also during out-patient care, to identify special therapy needs in these areas [8]. If relevant impairments are found, more extensive diagnostic tests are necessary in order to evaluate the impairments in depth and to plan the therapeutic intervention accordingly. Follow-up diagnostics of the three functional areas should be performed 2–4 weeks after hospital discharge and repeated regularly, at least 6–12 months after the end of inpatient rehabilitation [8].

### Physical rehabilitation

#### Early mobilization

*Research question* Does early mobilization of critically ill patients reduce the incidence or duration of PICS?

*Recommendation 1* Early mobilization ought to be started within the first few days in the ICU, depending on the patient's resilience and general condition.

**Table:** 

Grade of recommendation: **A**	Level of evidence: OECBM 1	Quality of evidence: High Selected references: [28–36]

Therapy Option 1: Supplemental use of ergometers (bed cycling) in addition to early mobilization can be considered.

**Table:** 

Grade of recommendation: **0**	Level of evidence:	OECBM 1	Quality of evidence: High Selected references: [35, 36]

Rationale Mobilization is an energy-consuming process aimed at maintaining and promoting a person's mobility [37, 38]. Early mobilization is started within 72 h after admission [38] and intensified during the stay in ICU. Various methods of early mobilization can be performed with different approaches [39], including passive mobilization (bed mobility, neuromuscular electrical stimulation (NMES)), assisted exercises (bed cycling, robotics, functional exercises, resistance exercises, transfers), active exercises (active exercises, activities of daily living, walking), or other exercises, e.g., cognitive exercises. Early mobilization is generally recommended during the stay on ICU, as it can have positive effects on the duration of ventilation and length of stay, delirium incidence, and muscle strength at the time of discharge [33]. Although early mobilization in the ICU does not seem to have a significant effect on long-term physical, functional, cognitive, or psychosocial outcome compared to usual care, short-term effects such as reduction of mechanical ventilation and length of stay or delirium frequency in the ICU can be demonstrated. These issues are highly relevant for patients and families. Therefore, we recommend structured, interprofessional implementation of early mobilization of critically ill patients according to defined inclusion and exclusion criteria with the best possible dosage and frequency.

#### Physical therapy

*Research question* Which physical therapy approach can reduce typical manifestations of PICS such as intensive care unit-acquired weakness (ICU-AW)?

Therapy Option 2: Wheelchair cycle ergometer training can be used in addition to standard physical therapy to improve muscle strength and cardiovascular fitness.

**Table:** 

Grade of recommendation: **0**	Level of evidence: OECBM 2	Quality of evidence: Low Selected reference: [40]

Therapy Option 3: Strength training can be used as an adjunct to standard physical therapy to increase walking speed.

**Table:** 

Grade of recommendation: **0**	Level of evidence: OECBM 2	Quality of evidence: Low Selected reference: [40]

Therapy Option 4: Electrical stimulation of the ventral thigh musculature can be used to strengthen the muscles.

**Table:** 

Grade of recommendation: **0**	Level of evidence: OECBM 2	Quality of evidence: Low Selected reference: [41]

*Recommendation 2* Training of the inspiratory muscles using an inhalation trainer should be used to increase the strength of the inspiratory muscles and the quality of life in the short term as an adjunct to standard physical therapy.

**Table:** 

Grade of recommendation: **B**	Level of evidence: OECBM 2	Quality of evidence: Moderate Selected reference: [42]

*Rationale* Patients with PICS often suffer from ICU-AW, leading to limitations in body functions and activities as well as reduced quality of life [24, 43, 44]. Motor rehabilitation therapy plays an essential role in the treatment of these patients and the prevention of further complications [45]. Motor rehabilitation begins with the diagnosis of motor impairment and continues after hospital discharge. Interventions such as bed- and wheelchair ergometer training, functional electrical stimulation, (inspiratory) muscle training, and outpatient physical therapy programs were evaluated and compared to standard therapy.

There were no statistically significant differences compared to the respective standard therapy [36, 40–42, 46]. Device-assisted therapy can facilitate the early mobilization and physical rehabilitation of critically ill patients and can complement conventional physiotherapy and occupational therapy with the aim of improving sensorimotor function (arm, hand, stance, and gait function) and cardiopulmonary exercise capacity [47]. In the current absence of scientific evidence, we cannot make recommendations for device-assisted therapy for patients with PICS. In clinical practice, a robot-assisted tilt table with and without electrical stimulation, robot-assisted movement training (bed cycling), robot-assisted-standing, and gait training are increasingly used.

#### Dysphagia and removal of tracheostomy tubes

*Research question* Which speech-language therapy (SLT) interventions can lead to removal of tracheostomy tubes and improvement of swallowing patients with PICS?

*Recommendation 3* As dysphagia is frequent in patients with tracheostomy, standardized assessment of swallowing function should be performed before oral nourishment is initiated.

**Table:** 

Grade of recommendation: **B**	Level of evidence: OECBM 1	Quality of evidence: High Selected references: [48–54]

*Rationale* Dysphagia is common in patients with tracheostomy [54]. An important task for SLTs is diagnosis and treatment of swallowing disorders as well as tracheostomy tube management culminating in removal of tracheostomy tubes.

No studies were concerned specifically with PICS and dysphagia therapy; therefore, no recommendations regarding dysphagia therapy for patients with PICS can be made. However, there are strong resemblances with other severely affected critically ill patients in neurological early rehabilitation. Thus, recommendations of the respective guidelines [55] for these cases can be considered valid for patients with PICS.

Clinical assessment of swallowing with a tracheostomy tube with deflated cuff and speaking valve for bedside screenings has low reliability [49, 51]. Penetrations cannot be reliably identified by the Evans-Blue-Test [53]. Fiberoptic endoscopic evaluation of swallowing (FEES) can be performed at the bedside in patients with cognitive and/or motor impairment [48]. Methods of removing a tracheostomy tube vary greatly [52]. FEES before removal of tracheostomy tube improves outcome [50].

### Cognitive rehabilitation

For cognitive rehabilitation, four recommendations were developed.

#### Cognitive therapies

*Research question* Which cognitive interventions can prevent development of PICS or reduce its manifestation?

*Recommendation 4* Computer-based learning for attention functions and/ or therapy aiming at improvement of cognition should be performed with critically ill patients in the ICU and during further rehabilitation.

**Table:** 

Grade of recommendation: **B**	Level of evidence: OECBM 1, 2	Quality of evidence: Low. Selected references: [56–58]

*Rationale* Patients with PICS suffer from acute and chronic impairments of attention, memory, and executive functions. Scoping reviews including the RCT of Jackson et al. [57] found significant effects of cognitive interventions (training of attention functions, psychoeducation, goal-management training) on scores in MMSE, MoCA, or TL-D and on quality of life outcomes [53, 55]. A 3-month study compared patients discharged from ICU given out-patient cognitive training in combination with physiotherapy and psychoeducation with patients on a waiting list, with follow-up after another 3 months [54]. The intervention group showed significant improvements in executive functions.

#### Delirium prevention and therapy

*Research question 1* Which non-pharmacological interventions can prevent delirium in critically ill patients?

*Recommendation 5* Interventions for delirium prophylaxis ought to include multimodal sensory, cognitive, and emotional stimulation (mobilization, purposeful stimulation and engagement, aids for orientation, contact with family members).

**Table:** 

Grade of recommendation: **A**	Evidence level: OECBM Level 1	Quality of evidence: High Selected references: [59, 60]

*Recommendation *6 Interventions for reduction of stress (pain, anxiety, sleep, noise), improvement of communication, and family care should be taken.

**Table:** 

Grade of recommendation: **B**	Level of evidence: OECBM Level 2	Quality of evidence: low Selected references: [59, 61–63]

*Rationale* A systematic review analyzed 21 studies on delirium prevention. They recommend measures such as the ABCDEF-bundle combined with regular delirium assessments with e.g., the Confusion Assessment Method for the ICU (CAM-ICU) or the Intensive Care Delirium Screening Checklist (ICDSC) [58]. Delirium prevention in critically ill patients requires specially trained teams and multimodal interventions [59]. There are indications that stressors (such as pain, hunger, thirst, catheters, infusion systems, isolation, disorientation, anxiety, lack of sleep) facilitate the development of delirium. Besides their cognitive and physical impairments, critically ill patients suffer from ICU-specific problems (noise of monitors, commotion, isolation) and concurrent symptoms such as hunger, thirst, pain, anxiety, dyspnea, and depression, which affect and aggravate the symptoms of PICS. Communications aids (letter boards, tablets, tracheostomy tubes with speaking valves) contribute to delirium prevention. Early mobilization (depending on patients’ abilities from sitting on the edge of the bed to walking with the therapist) and contact with family members significantly reduce the incidence of delirium [60]. The meta-analysis of Deng et al. [63] of studies with a total of 6499 critically ill patients indicates reduced rates of delirium from contact with relatives and multimodal interventions (control of concurrent symptoms, stress reduction). Early mobilization results in a significant reduction of mortality. Liang et al. [60] and Litton et al. [62] found for 7159 and 1455 included critically ill patients, respectively, positive effects for cognitive programs (orientation aids, improvement of communication) and environment modification (noise reduction, ear-plugs, eye shields, light management) on frequency and duration of delirium as well as on mortality. Delirium increases the probability of developing PICS. Therapeutic interventions are limited once delirium has evolved. Thus, delirium prevention may prevent or reduce the symptoms of PICS.

*Research question 2* How efficient is treatment with haloperidol vs placebo in the prevention or treatment of delirium?

*Recommendation 7* A prophylactic treatment with haloperidol for ventilated patients should not be implemented, as comparison to placebo shows no effect in incidence, severity, duration, or outcome of delirium.

**Table:** 

Grade of recommendation: **B**	Level of evidence: OECBM Level	Quality of evidence: High Selected references: [64, 65]

*Rationale* Studies on pharmacological interventions compared the effect of haloperidol versus placebo on incidence, duration, and outcome of delirium and time required for weaning. A systematic review [64] and a qualitative synthesis [65] found no significant differences between haloperidol and placebo with respect to delirium incidence, severity, duration, and outcome.

*Research demand*:

### Psychological rehabilitation

#### Psychotherapy

*Research question* Can psychological interventions counteract the psychological sequalae of PICS (anxiety, depression, post-traumatic stress)?

*Recommendation 8* Critically ill patients with adaptation disorders such as anxiety and depression benefit from psychological interventions. These should be offered in the ICU and/or early rehabilitation and also to family members, if possible.

**Table:** 

Grade of recommendation: **B**	Level of evidence: OECBM 2	Quality of evidence: Low Selected reference: [66]

*Recommendation *9 Post-traumatic stress reactions should be treated by interventions such as psychoeducation and psychotherapy.

**Table:** 

Grade of recommendation: **B**	Level of evidence: OECBM 2	Quality of evidence: Low Selected reference: [67]

*Recommendation 10* Access to professional support and follow-up-care targeting psychological stabilization should be offered in the first 12 months after discharge.

**Table:** 

Grade of recommendation: **B**	Level of evidence: OECBM 2	Quality of evidence: Low Selected reference: [68]

*Rationale* Critically ill patients are at risk for psychological/mental disorders. Anxiety and depression are common for months after discharge [69]. We identified two RCTs [66, 67] and three reviews [70, 71], one of which is a Cochrane report [68]. In the ICU a multidisciplinary approach is recommended, including mobilization, facilitation of communication, information, and resilience training. After discharge, psychotherapeutic interventions should be offered [72] and patients referred to specialized services [68]. The systematic reviews identified only a PTSD-reducing effect [68]. Resilience training was able to reduce anxiety and depression in the intervention group, an effect stable over 12 weeks [66]; Peris et al. [67] found a significant reduction of PTSD symptoms and a significant improvement of psychological symptoms and stabilization of mental health.

#### ICU diaries

*Research question* Can ICU diaries reduce the incidence of symptoms of anxiety, depression, and PTSD in critically ill patients after discharge from the ICU?

*Recommendation 11* ICU diaries ought to be implemented to reduce the risks of symptoms of anxiety, depression, and PTSD in critically ill patients after discharge from the ICU.

**Table:** 

Grade of recommendation: **A**	Level of evidence: OECBM 1	Quality of evidence: Moderate. Selected references: [73, 74]

*Recommendation 12* In post-ICU follow-up, ICU diaries ought to be read with health care professionals.

**Table:** 

Grade of recommendation: **A**	Level of evidence: OECBM 1	Quality of evidence: Moderate: Selected reference: [73]

*Rationale* An ICU-diary is written by nurses, therapists, or family members to record events about the period that critically ill patients usually cannot remember. It may contain photographs and psychoeducational information in addition to handwritten entries about events, visits, or patient progress. In general, use of ICU diaries reduces the risks of anxiety, depressive symptoms, and PTSD. Patient-centered editing of ICU diaries with persons trained for this purpose can be important. Systematic meta-syntheses indicate a generally positive reception by critically ill patients and family members [75], who express outcomes such as better understanding of what was experienced, effective coping, continuation of relationship building, meaningfulness, and other benefits.

## Discussion

One statement 12 recommendations, and four therapy options for the rehabilitation of critically ill patients with PICS could be extracted from the literature. The recommendations are categorized by the three domains of impairment: Physical Rehabilitation, Cognitive Rehabilitation, and Psychological Rehabilitation.

Epidemiological studies report PICS affects 50–70% of intensive care unit survivors [3], and its effects can persist for 5–15 years after ICU hospitalization [76]. PICS is a neurologically heterogenous syndrome that affects patients weeks to months after discharge from the ICU and manifests with impairments in at least one of the following three domains: physical, cognitive, and psychological health.

This is the first guideline on multimodal rehabilitative therapies for patients affected by PICS based on the critical appraisal of the present scientific evidence. A multidisciplinary task force addressed 10 principal research questions pertaining to the rehabilitative therapy of PICS and searched for the best available evidence, determined its quality, and formulated therapy recommendations. The task force extracted four strong recommendations, eight recommendations and 4 therapy options. The 4 strong recommendations address the rehabilitation of all three domains, early mobilization, the usage of ICU diaries, and the prevention and treatment of delirium, respectively. The four treatment options all refer to the therapy for physical impairments. The previous Cochrane review by Mehrholz [77] also did not yield any recommendations regarding the treatment of ICU-AW. Two research questions regarding the therapy to reduce PICS-related fatigue and the therapy to ensure the return to work, remain unanswered due to the absence of appropriate scientific evidence. The various manifestations of PICS regarding their quality and time of appearance may explain the paucity of scientific evidence. In addition, the heterogeneity of studies relating to the patient populations, assessments, and outcome measures do not allow comparison of the therapies. There are only few systematic reviews including homogenous RCTs. Thus there is a tremendous need for further randomized, controlled studies comparing different interventions as we indicate the specific research demands in Table 3.

**Table 3:** Future research demands categorized by the three domains of impairment: physical rehabilitation, cognitive rehabilitation, and psychological rehabilitation

*Diagnosis of PICS*
Which combination and timing of assessments are most valid, reliable, and feasible for detection, report and evaluation of different symptoms of PICS in patients who survive critical illness?
*Rehabilitation of physical health*
*Early mobilization* Which patients require what type of early mobilization, and how should its intensity be adapted during rehabilitation? What impact does early rehabilitation have on long-term outcomes? Does early mobilization prevent or reduce specific symptoms of PICS? What is the impact of pre-existing frailty on long-term outcome after discharge from ICU?
*Physical therapy* What length and frequency of interventions (i.e. strength training etc.) optimize potential effects? Is device-assisted physical therapy (i.e. wheelchair cycle ergometers, electrical stimulation etc.) effective for specific subgroups of patients with PICS?
*Speech-Language-Therapy* Do interventions, such as swallowing assessments, FEES, tracheostomy tube management, swallowing therapy lead to improvements of physical symptoms typical for PICS such as diminished coordination of respiration, swallowing and coughing, and/or swallowing function?
*Rehabilitation of cognitive health*
*Cognitive therapy* Do cognitive therapies (training of attention, computer-based training psychoeducation, virtual reality, goal management training) improve attention, memory, and executive functions in patients with PICS and those at risk for PICS?
*Non-pharmacological delirium prevention and therapy* Effect of non-pharmacological interventions (i.e. stress reduction, pain reduction, reduction of sleep deprivation) versus standard or no therapy on cognitive functions or reduction of cognitive PICS symptoms, activities, and participation
*Pharmacological delirium prevention and therapy* Effects of pharmacological interventions vs standard or no therapy on delirium reduction regarding incidence, duration, and cognitive outcome
*Rehabilitation of psychological health*
*Psychotherapy*:When does psychotherapy improve psychological symptoms typical for PICS such as anxiety, depression, and traumatization?
*ICU-diary* When is the best time to read diaries, how to reach non-responders/patients who avoid reading their diary, and is there a different impact of diaries written by families compared to those written by healthcare professionals

*Abbreviations*: *FEES* flexible endoscopic evaluation of swallowing, *ICU* intensive care unit, *PICS* post intensive care syndrome

Symptoms of PICS can occur in patients with critical illness at any time; as early as 48 h post admission to the ICU, masked by sedation during the stay in the ICU or they can ensue delayed during in-patient rehabilitation or even when patients are already discharged home. In addition, different symptoms can manifest simultaneously or at different phases of the critical illness. Therefore, it is important to screen repeatedly for impairments of physical/motorsensory, cognitive and psychological functions. Although the awareness towards PICS is growing internationally, care for critical illness survivors is still fragmented. Chronic health impairments require continuity of care. Similarly as for people with stroke, it is well-understood that the best outcome is achieved with a multi-stage rehabilitation pathway [78]. PICS-rehabilitation should also occur in various health care settings from the intensive care unit, the acute rehabilitation unit, post-acute rehabilitation unit, to the outpatient clinic, community-based, and domiciliary settings. There is an urgent need to promote, achieve and sustain multidisciplinary rehabilitative therapy for patients affected by PICS. This is best performed by a multidisciplinary approach involving specialized doctors, nurses, and therapists from various disciplines with the best available external evidence being implemented in clinical practice.

### Limitations

This guideline has several limitations. The very specific literature search considered RCTs with more than 10 participants and systematic reviews; a less restrictive search could have yielded more results; however, the risk of bias and the validity of the studies would have been reduced, thus it is unlikely that more or different recommendations would have been obtained. PICS-Family and other family aspects were not considered, although there is often an interaction between patients and families regarding PICS. The Guideline for Family-Centered Care is currently under revision, and the results should be considered in the next update of the present guideline. Further limitations are the lack of recommendations on fatigue and social aspects such as quality of life and return to work, as there is currently no robust evidence available and therefore no recommendations. Likewise, there are few studies validating various assessments for diagnosing PICS and its sequelae, yet they are no ideal randomised controlled clinical trials, not comparable and do not allow to extrapolate recommendations. Finally, the following aspects could not be considered as the present guideline is the first to be published on multimodal rehabilitative therapies for patients affected by PICS: description of management options; population or clinical situation most appropriate to each option, facilitators and barriers to the guideline’s application, advices and/or tools on how the recommendations can be applied into practice, resource implications, or monitoring criteria. Clinical guidelines can help to maximize achievement of treatment goals. Yet the development of guidelines like the present one does not ensure their use. Further studies are needed to assess the feasibility and implementation of these guidelines in routine clinical practice.

## Conclusions

The appropriate rehabilitative therapy for patients with PICS remains a major challenge in routine clinical practice. An individualized, multimodal and interdisciplinary approach for the rehabilitative therapy, repetitive assessments of physical, psychological and cognitive health functions and adherence to evidence-based guidance may be key to improving patient outcomes, which future outcome studies may prove. As new evidence becomes available, this guideline will need to be updated accordingly.

## Supplementary Information

**Additional file 1. Table S1**: Authors’ Involvement. **Table S2**: AGREE Reporting Checklist.

## Data Availability

All data generated or analysed during this study are included in this published article and its supplementary information files.

## References

[1] Kleinpell R, Grabenkort WR, Boyle WA, Vines DL, Olsen KM (2021). The society of critical care medicine at 50 years: interprofessional practice in critical care: looking back and forging ahead. Crit Care Med.

[2] Ramnarain D, Aupers E, den Oudsten B, Oldenbeuving A, de Vries J, Pouwels S (2021). Post Intensive Care Syndrome (PICS): an overview of the definition, etiology, risk factors, and possible counseling and treatment strategies. Expert Rev Neurother.

[3] Needham DM, Davidson J, Cohen H, Hopkins RO, Weinert C, Wunsch H, Zawistowski C, Bemis-Dougherty A, Berney SC, Bienvenu OJ, Brady SL, Brodsky MB, Denehy L, Elliott D, Flatley C, Harabin AL, Jones C, Louis D, Meltzer W, Muldoon SR, Palmer JB, Perme C, Robinson M, Schmidt DM, Scruth E, Spill GR, Storey CP, Render M, Votto J, Harvey MA (2012). Improving long-term outcomes after discharge from intensive care unit: report from a stakeholders' conference. Crit Care Med.

[4] Rousseau AF, Minguet P, Colson C, Kellens I, Chaabane S, Delanaye P, Cavalier E, Chase JG, Lambermont B, Misset B (2021). Post-intensive care syndrome after a critical COVID-19: cohort study from a Belgian follow-up clinic. Ann Intensive Care.

[5] Fan E, Cheek F, Chlan L, Gosselink R, Hart N, Herridge MS, Hopkins RO, Hough CL, Kress JP, Latronico N, Moss M, Needham DM, Rich MM, Stevens RD, Wilson KC, Winkelman C, Zochodne DW, Ali NA (2014). An official American Thoracic Society Clinical Practice guideline: the diagnosis of intensive care unit-acquired weakness in adults. Am J Respir Crit Care Med.

[6] Bienvenu OJ, Neufeld KJ, Needham DM (2012). Treatment of four psychiatric emergencies in the intensive care unit. Crit Care Med.

[7] Brown SM, Bose S, Banner-Goodspeed V, Beesley SJ, Dinglas VD, Hopkins RO, Jackson JC, Mir-Kasimov M, Needham DM, Sevin CM (2019). Approaches to Addressing post-intensive care syndrome among intensive care unit survivors. A narrative review. Ann Am Thorac Soc.

[8] Mikkelsen ME, Still M, Anderson BJ, Bienvenu OJ, Brodsky MB, Brummel N, Butcher B, Clay AS, Felt H, Ferrante LE, Haines KJ, Harhay MO, Hope AA, Hopkins RO, Hosey M, Hough CTL, Jackson JC, Johnson A, Khan B, Lone NI, MacTavish P, McPeake J, Montgomery-Yates A, Needham DM, Netzer G, Schorr C, Skidmore B, Stollings JL, Umberger R, Andrews A, Iwashyna TJ, Sevin CM (2020). Society of critical care medicine's international consensus conference on prediction and identification of long-term impairments after critical illness. Crit Care Med.

[9] Vester LB, Holm A, Dreyer P (2022). Patients' and relatives' experiences of post-ICU everyday life: a qualitative study. Nurs Crit Care.

[10] Marra A, Pandharipande PP, Girard TD, Patel MB, Hughes CG, Jackson JC, Thompson JL, Chandrasekhar R, Ely EW, Brummel NE (2018). Co-occurrence of post-intensive care syndrome problems among 406 survivors of critical illness. Crit Care Med.

[11] Rawal G, Yadav S, Kumar R (2017). Post-intensive care syndrome: an overview. J Transl Int Med.

[12] Dinglas VD, Faraone LN, Needham DM (2018). Understanding patient-important outcomes after critical illness: a synthesis of recent qualitative, empirical, and consensus-related studies. Curr Opin Crit Care.

[13] Inoue S, Hatakeyama J, Kondo Y, Hifumi T, Sakuramoto H, Kawasaki T, Taito S, Nakamura K, Unoki T, Kawai Y, Kenmotsu Y, Saito M, Yamakawa K, Nishida O (2019). Post-intensive care syndrome: its pathophysiology, prevention, and future directions. Acute Med Surg.

[14] Pandharipande PP, Girard TD, Jackson JC, Morandi A, Thompson JL, Pun BT, Brummel NE, Hughes CG, Vasilevskis EE, Shintani AK, Moons KG, Geevarghese SK, Canonico A, Hopkins RO, Bernard GR, Dittus RS, Ely EW (2013). Long-term cognitive impairment after critical illness. N Engl J Med.

[15] Parker AM, Sricharoenchai T, Raparla S, Schneck KW, Bienvenu OJ, Needham DM (2015). Posttraumatic stress disorder in critical illness survivors: a metaanalysis. Crit Care Med.

[16] Nikayin S, Rabiee A, Hashem MD, Huang M, Bienvenu OJ, Turnbull AE, Needham DM (2016). Anxiety symptoms in survivors of critical illness: a systematic review and meta-analysis. Gen Hosp Psychiatry.

[17] Rabiee A, Nikayin S, Hashem MD, Huang M, Dinglas VD, Bienvenu OJ, Turnbull AE, Needham DM (2016). Depressive symptoms after critical illness: a systematic review and meta-analysis. Crit Care Med.

[18] Hatch R, Young D, Barber V, Griffiths J, Harrison DA, Watkinson P (2018). Anxiety, depression and post traumatic stress disorder after critical illness: a UK-wide prospective cohort study. Crit Care.

[19] Platz T (2021). Evidence-based Practice Guidelines for the German Society for Neurology (DGN) and the German Society for Neurorehabilitation (DGNR): methods for systematic evidence-to-decision process. Fortschr Neurol Psychiatr.

[20] OCEBM Levels of Evidence Working Group (2011) The Oxford 2011 Levels of Evidence. In: Editor (ed)^(eds) Book The Oxford 2011 Levels of Evidence. City, pp.

[21] Balshem H, Helfand M, Schünemann HJ, Oxman AD, Kunz R, Brozek J, Vist GE, Falck-Ytter Y, Meerpohl J, Norris S, Guyatt GH (2011). GRADE guidelines: 3. Rating the quality of evidence. J Clin Epidemiol.

[22] Brouwers MC, Kerkvliet K, Spithoff K (2016). The AGREE Reporting Checklist: a tool to improve reporting of clinical practice guidelines. BMJ.

[23] Needham DM, Sepulveda KA, Dinglas VD, Chessare CM, Friedman LA, Bingham CO, Turnbull AE (2017). Core outcome measures for clinical research in acute respiratory failure survivors. an international modified Delphi consensus study. Am J Respir Crit Care Med.

[24] Van Aerde N, Meersseman P, Debaveye Y, Wilmer A, Gunst J, Casaer MP, Bruyninckx F, Wouters PJ, Gosselink R, Van den Berghe G, Hermans G (2020). Five-year impact of ICU-acquired neuromuscular complications: a prospective, observational study. Intensive Care Med.

[25] Yuan C, Timmins F, Thompson DR (2022). Post-intensive care syndrome: time for a robust outcome measure?. Nurs Crit Care.

[26] Jeong YJ, Kang J (2019). Development and validation of a questionnaire to measure post-intensive care syndrome. Intensive Crit Care Nurs.

[27] Spies CD, Krampe H, Paul N, Denke C, Kiselev J, Piper SK, Kruppa J, Grunow JJ, Steinecke K, Gülmez T, Scholtz K, Rosseau S, Hartog C, Busse R, Caumanns J, Marschall U, Gersch M, Apfelbacher C, Weber-Carstens S, Weiss B (2021). Instruments to measure outcomes of post-intensive care syndrome in outpatient care settings—results of an expert consensus and feasibility field test. J Intensive Care Soc.

[28] Taito S, Taito M, Banno M, Tsujimoto H, Kataoka Y, Tsujimoto Y (2018). Rehabilitation for patients with sepsis: a systematic review and meta-analysis. PLoS ONE.

[29] Eggmann S, Verra ML, Luder G, Takala J, Jakob SM (2018). Effects of early, combined endurance and resistance training in mechanically ventilated, critically ill patients: a randomised controlled trial. PLoS ONE.

[30] Fuke R, Hifumi T, Kondo Y, Hatakeyama J, Takei T, Yamakawa K, Inoue S, Nishida O (2018). Early rehabilitation to prevent postintensive care syndrome in patients with critical illness: a systematic review and meta-analysis. BMJ Open.

[31] Wright SE, Thomas K, Watson G, Baker C, Bryant A, Chadwick TJ, Shen J, Wood R, Wilkinson J, Mansfield L, Stafford V, Wade C, Furneval J, Henderson A, Hugill K, Howard P, Roy A, Bonner S, Baudouin S (2018). Intensive versus standard physical rehabilitation therapy in the critically ill (EPICC): a multicentre, parallel-group, randomised controlled trial. Thorax.

[32] Takaoka A, Utgikar R, Rochwerg B, Cook DJ, Kho ME (2020). The efficacy and safety of in-intensive care unit leg-cycle ergometry in critically ill adults. a systematic review and meta-analysis. Ann Am Thorac Soc.

[33] Waldauf P, Jiroutková K, Krajčová A, Puthucheary Z, Duška F (2020). Effects of rehabilitation interventions on clinical outcomes in critically ill patients: systematic review and meta-analysis of randomized controlled trials. Crit Care Med.

[34] Wang W, Xu C, Ma X, Zhang X, Xie P (2020). Intensive care unit-acquired weakness: a review of recent progress with a look toward the future. Front Med (Lausanne).

[35] Berney S, Hopkins RO, Rose JW, Koopman R, Puthucheary Z, Pastva A, Gordon I, Colantuoni E, Parry SM, Needham DM, Denehy L (2021). Functional electrical stimulation in-bed cycle ergometry in mechanically ventilated patients: a multicentre randomised controlled trial. Thorax.

[36] Waldauf P, Hrušková N, Blahutova B, Gojda J, Urban T, Krajčová A, Fric M, Jiroutková K, Řasová K, Duška F (2021). Functional electrical stimulation-assisted cycle ergometry-based progressive mobility programme for mechanically ventilated patients: randomised controlled trial with 6 months follow-up. Thorax.

[37] Amidei C (2012). Mobilisation in critical care: a concept analysis. Intensive Crit Care Nurs.

[38] Bein T (2015). Move to improve: new guidelines on positional therapy and early mobilization. Anaesthesist.

[39] Clarissa C, Salisbury L, Rodgers S, Kean S (2019). Early mobilisation in mechanically ventilated patients: a systematic integrative review of definitions and activities. J Intensive Care.

[40] Veldema J, Bösl K, Kugler P, Ponfick M, Gdynia HJ, Nowak DA (2019). Cycle ergometer training versus resistance training in ICU-acquired weakness. Acta Neurol Scand.

[41] Chen YH, Hsiao HF, Li LF, Chen NH, Huang CC (2019). Effects of electrical muscle stimulation in subjects undergoing prolonged mechanical ventilation. Respir Care.

[42] Bissett BM, Leditschke IA, Neeman T, Boots R, Paratz J (2016). Inspiratory muscle training to enhance recovery from mechanical ventilation: a randomised trial [with consumer summary]. Thorax.

[43] Saccheri C, Morawiec E, Delemazure J, Mayaux J, Dubé BP, Similowski T, Demoule A, Dres M (2020). ICU-acquired weakness, diaphragm dysfunction and long-term outcomes of critically ill patients. Ann Intensive Care.

[44] Intiso D, Centra AM, Bartolo M, Gatta MT, Gravina M, Di Rienzo F (2022). Recovery and long term functional outcome in people with critical illness polyneuropathy and myopathy: a scoping review. BMC Neurol.

[45] Hodgson CL, Tipping CJ (2017). Physiotherapy management of intensive care unit-acquired weakness. J Physiother.

[46] Connolly B, Thompson A, Douiri A, Moxham J, Hart N (2015). Exercise-based rehabilitation after hospital discharge for survivors of critical illness with intensive care unit-acquired weakness: a pilot feasibility trial. J Crit Care.

[47] Atashzar SF, Carriere J, Tavakoli M (2021). Review: how can intelligent robots and smart mechatronic modules facilitate remote assessment, assistance, and rehabilitation for isolated adults with neuro-musculoskeletal conditions?. Front Robot AI.

[48] Langmore SE, Kenneth SMA, Olsen N (1988). Fiberoptic endoscopic examination of swallowing safety: a new procedure. Dysphagia.

[49] Hales PA, Drinnan MJ, Wilson JA (2008). The added value of fibreoptic endoscopic evaluation of swallowing in tracheostomy weaning. Clin Otolaryngol.

[50] Warnecke T, Suntrup S, Teismann IK, Hamacher C, Oelenberg S, Dziewas R (2013). Standardized endoscopic swallowing evaluation for tracheostomy decannulation in critically ill neurologic patients. Crit Care Med.

[51] Lynch YT, Clark BJ, Macht M, White SD, Taylor H, Wimbish T, Moss M (2017). The accuracy of the bedside swallowing evaluation for detecting aspiration in survivors of acute respiratory failure. J Crit Care.

[52] Singh RK, Saran S, Baronia AK (2017). The practice of tracheostomy decannulation—a systematic review. J Intensive Care.

[53] Linhares Filho TA, Arcanjo FPN, Zanin LH, Portela HA, Braga JM, da Luz PV (2019). The accuracy of the modified Evan's blue dye test in detecting aspiration in tracheostomised patients. J Laryngol Otol.

[54] Skoretz SA, Riopelle SJ, Wellman L, Dawson C (2020). Investigating swallowing and tracheostomy following critical illness: a scoping review. Crit Care Med.

[55] Dziewas R, Pflug C (2020) Neurogene Dysphagie, S1-Leitlinie. In: Editor (ed)^(eds) Book Neurogene Dysphagie, S1-Leitlinie. City, pp.

[56] Geense WW, van den Boogaard M, van der Hoeven JG, Vermeulen H, Hannink G, Zegers M (2019). Nonpharmacologic interventions to prevent or mitigate adverse long-term outcomes among ICU survivors: a systematic review and meta-analysis. Crit Care Med.

[57] Jackson JC, Ely EW, Morey MC, Anderson VM, Denne LB, Clune J, Siebert CS, Archer KR, Torres R, Janz D, Schiro E, Jones J, Shintani AK, Levine B, Pun BT, Thompson J, Brummel NE, Hoenig H (2012). Cognitive and physical rehabilitation of intensive care unit survivors: results of the RETURN randomized controlled pilot investigation. Crit Care Med.

[58] Muradov O, Petrovskaya O, Papathanassoglou E (2021). Effectiveness of cognitive interventions on cognitive outcomes of adult intensive care unit survivors: a scoping review. Aust Crit Care.

[59] Devlin JW, Skrobik Y, Gélinas C, Needham DM, Slooter AJC, Pandharipande PP, Watson PL, Weinhouse GL, Nunnally ME, Rochwerg B, Balas MC, van den Boogaard M, Bosma KJ, Brummel NE, Chanques G, Denehy L, Drouot X, Fraser GL, Harris JE, Joffe AM, Kho ME, Kress JP, Lanphere JA, McKinley S, Neufeld KJ, Pisani MA, Payen JF, Pun BT, Puntillo KA, Riker RR, Robinson BRH, Shehabi Y, Szumita PM, Winkelman C, Centofanti JE, Price C, Nikayin S, Misak CJ, Flood PD, Kiedrowski K, Alhazzani W (2018). Clinical practice guidelines for the prevention and management of pain, agitation/sedation, delirium, immobility, and sleep disruption in adult patients in the ICU. Crit Care Med.

[60] Liang S, Chau JPC, Lo SHS, Zhao J, Choi KC (2021). Effects of nonpharmacological delirium-prevention interventions on critically ill patients' clinical, psychological, and family outcomes: a systematic review and meta-analysis. Aust Crit Care.

[61] Trogrlić Z, van der Jagt M, Bakker J, Balas MC, Ely EW, van der Voort PH, Ista E (2015). A systematic review of implementation strategies for assessment, prevention, and management of ICU delirium and their effect on clinical outcomes. Crit Care.

[62] Litton E, Carnegie V, Elliott R, Webb SA (2016). The efficacy of earplugs as a sleep hygiene strategy for reducing delirium in the ICU: a systematic review and meta-analysis. Crit Care Med.

[63] Deng LX, Cao L, Zhang LN, Peng XB, Zhang L (2020). Non-pharmacological interventions to reduce the incidence and duration of delirium in critically ill patients: a systematic review and network meta-analysis. J Crit Care.

[64] Herling SF, Greve IE, Vasilevskis EE, Egerod I, Bekker Mortensen C, Møller AM, Svenningsen H, Thomsen T (2018). Interventions for preventing intensive care unit delirium in adults. Cochrane Database Syst Rev.

[65] Igwe EO, Nealon J, Mohammed M, Hickey B, Chou K-R, Chen K-H, Traynor V (2020). Multi-disciplinary and pharmacological interventions to reduce post-operative delirium in elderly patients: a systematic review and meta-analysis. J Clin Anesth.

[66] Vranceanu AM, Bannon S, Mace R, Lester E, Meyers E, Gates M, Popok P, Lin A, Salgueiro D, Tehan T, Macklin E, Rosand J (2020). Feasibility and efficacy of a resiliency intervention for the prevention of chronic emotional distress among survivor-caregiver dyads admitted to the neuroscience intensive care unit: a randomized clinical trial. JAMA Netw Open.

[67] Peris A, Bonizzoli M, Iozzelli D, Migliaccio ML, Zagli G, Bacchereti A, Debolini M, Vannini E, Solaro M, Balzi I, Bendoni E, Bacchi I, Trevisan M, Giovannini V, Belloni L (2011). Early intra-intensive care unit psychological intervention promotes recovery from post traumatic stress disorders, anxiety and depression symptoms in critically ill patients. Crit Care.

[68] Schofield-Robinson OJ, Lewis SR, Smith AF, McPeake J, Alderson P (2018). Follow-up services for improving long-term outcomes in intensive care unit (ICU) survivors. Cochrane Database Syst Rev.

[69] Svenningsen H, Langhorn L, Ågård AS, Dreyer P (2017). Post-ICU symptoms, consequences, and follow-up: an integrative review. Nurs Crit Care.

[70] Mehlhorn J, Freytag A, Schmidt K, Brunkhorst FM, Graf J, Troitzsch U, Schlattmann P, Wensing M, Gensichen J (2014). Rehabilitation interventions for postintensive care syndrome: a systematic review. Crit Care Med.

[71] Wade DF, Moon Z, Windgassen SS, Harrison AM, Morris L, Weinman JA (2016). Non-pharmacological interventions to reduce ICU-related psychological distress: a systematic review. Minerva Anestesiol.

[72] Kang J, Jeong YJ (2018). Embracing the new vulnerable self: a grounded theory approach on critical care survivors' post-intensive care syndrome. Intensive Crit Care Nurs.

[73] Sayde GE, Stefanescu A, Conrad E, Nielsen N, Hammer R (2020). Implementing an intensive care unit (ICU) diary program at a large academic medical center: results from a randomized control trial evaluating psychological morbidity associated with critical illness. Gen Hosp Psychiatry.

[74] Sun X, Huang D, Zeng F, Ye Q, Xiao H, Lv D, Zhao P, Cui X (2021). Effect of intensive care unit diary on incidence of posttraumatic stress disorder, anxiety, and depression of adult intensive care unit survivors: a systematic review and meta-analysis. J Adv Nurs.

[75] Barreto BB, Luz M, Alves Valente do Amaral Lopes S, Goulart Rosa R, Gusmao-Flores D (2021). Exploring patients' perceptions on ICU diaries: a systematic review and qualitative data synthesis. Crit Care Med.

[76] Desai SV, Law TJ, Needham DM (2011). Long-term complications of critical care. Crit Care Med.

[77] Mehrholz J, Pohl M, Kugler J, Burridge J, Mückel S, Elsner B (2015). Physical rehabilitation for critical illness myopathy and neuropathy. Cochrane Database Syst Rev.

[78] Platz T, Sandrini G (2020). Specialty grand challenge for neurorehabilitation research. Front Neurol.

